# Detection and Molecular Diversity of Spike Gene of Porcine Epidemic Diarrhea Virus in China

**DOI:** 10.3390/v5102601

**Published:** 2013-10-22

**Authors:** Jianfei Chen, Xiaozhen Liu, Da Shi, Hongyan Shi, Xin Zhang, Changlong Li, Yanbin Chi, Li Feng

**Affiliations:** Division of Swine Infectious Diseases, State Key Laboratory of Veterinary Biotechnology, Harbin Veterinary Research Institute, Chinese Academy of Agricultural Sciences, Harbin 150001, China

**Keywords:** PEDV, S gene, heterogeneity, genetic variation, variants

## Abstract

Since late 2010, porcine epidemic diarrhea virus (PEDV) has rapidly disseminated all over the China and caused considerable morbidity and high mortality (up to 100%) in neonatal piglets. 79.66% (141 of 177) pig farms in 29 provinces (excluding Tibet and Hainan, China) and 72.27% (417 of 577) samples were positive for PEDV confirmed by reverse transcription-polymerase chain reaction (RT-PCR). The full-length S genes of representative field strains were sequenced. 33 field strains share 93.5%–99.9% homologies with each other at the nucleotide sequence level and 92.3%–99.8% homologies with each other at the amino acids sequence level. Most field strains have nucleotide deletion and insertion regions, and show lower homologies (93.5%–94.2%) with Chinese classical strain CH/S, however higher homologies (97.1%–99.3%) with recent strain CHGD-1. The phylogenetic analysis showed there are classical strains and variants prevailing in pig herd in China. PEDV has a high detection rate in pig herds in China. Sequence analysis indicated the S genes of recent field strains have heterogeneity and the variants are predominant.

## 1. Introduction

Porcine epidemic diarrhea virus (PEDV) is a member of genus *Alphacoronavirus* within the family *Coronaviridae* and is a single-stranded positive-sense RNA virus. PEDV was firstly identified in Belgium in 1978 [1]. The disease caused by PEDV was firstly observed in feeder pigs and fattening swine in England [2] and was proposed the name “porcine epidemic diarrhea” (PED) [3]. PED is an acute and highly contagious enteric disease characterized by severe enteritis, vomiting and watery diarrhea in swine.

In China, PED was firstly occurred in Shanghai in 1973, however the causative agent was PEDV confirmed in 1984 [4]. Since first identified there have been occasional outbreaks of PEDV in China. However, outbreaks of PEDV have been observed on most swine breeding farms in most provinces since late 2010 in China. The piglets within one week of age and sometimes within only a few hours showed vomiting, severe watery diarrhea, severe dehydration and high mortality (up to 100%), but few sows or fattening swine showed any clinical signs during the outbreaks [5,6]. The economic loss caused by PEDV infection has been continuous and serious in China.

The Spike (S) gene is considered the most useful in revealing the genetic diversity of coronavirus isolates [7,8,9]. Like other coronaviruses, PEDV S gene also plays an important role in the molecular epidemiology and in the genetic variation of PEDV field strains [10,11,12]. In order to control and prevent PEDV infection, it is necessary for us to further investigate the prevalence of PEDV and the molecular characteristics of the S genes of Chinese PEDV field strains during 2011–2012. In this study, we firstly detected PEDV in the samples collected from 177 farms, then analyzed the sequence characteristics of S genes of field strains and performed the phylogenetic analysis of the field strains.

## 2. Results

### 2.1. PEDV Detection

79.66% (141 of 177) pig farms in 29 provinces (excluding Tibet and Hainan, China) and 72.27% (417 of 577) samples were positive for PEDV. 417 positive samples included 361 (71.63%; of 504) small intestine samples and 56 (76.71%; of 73) milk samples. 

### 2.2. Nucleotide and Amino Acid Sequence Analysis

The full-length nucleotide sequences of S genes of 33 field strains were determined to investigate their genetic characterization. The sequence data found that the S genes of recently Chinese field strains have 4,146–4,170 nucleotides (nt) in length (Table 1). Compared to the S gene (4152 nt) of CV777, the S genes of 3 field strains are 6 nt shorter and consist of 4,146 n, the S genes of 8 field strains are 3 nt shorter and consist of 4,149 nt, the S genes of 2 field strains consist of 4152 nt, the S genes of 2 field strains are 6 nt longer and consist of 4158 nt, the S genes of 17 field strains are 9 nt longer and consist of 4,161 nt and the S gene of 1 field strain are 18 nt longer consists of 4,170 nt. These consequences were due to the presences of the number of deleted or inserted nucleotides that were accumulated in the *N*-terminus of the S gene. 

**Table 1:** The recently Chinese PEDV field strains used in this study

Field strains	Abbreviations	Regions	S genes (nt)	S proteins (aa)	Accession numbers
CH/FJND-2/2011	FJND-2	Ningde, Fujian	4146	1381	JN315706
CH/GDQY/2011	GDQY	Qingyuan ,Guangdong	4146	1381	JN601051(S1);JQ638914(S2)
CH/GXWM/2011	GXWM	Wuming, Guangxi	4146	1381	JN601045(S1);JQ638911(S2)
CH/BJSY/2011	BJSY	Shunyi, Beijing	4149	1382	JQ638921
CH/FJND-1/2011	FJND-1	Ningde, Fujian	4149	1382	JN543367
CH/FJND-4/2011	FJND-4	Ningde, Fujian	4149	1382	JN601044(S1);JQ638506(S2)
CH/GXNN/2011	GXNN-1	Nanning, Guangxi	4149	1382	JN601049(S1);JQ638912(S2)
CH/HNZZ/2011	HNZZ	Zhengzhou, Henan	4149	1382	JN601050(S1);JQ638512(S2)
CH/JL/2011	JL	Jilin	4149	1382	JQ638924
CH/AHHF/2012	AHHF	Hefei, Anhui	4149	1382	JX018181
CH/YNKM/2012	YNKM	Kunming, Yunnan	4149	1382	JX018180
CH/JLGZL/2011	JLGZL	Siping, Jilin	4152	1383	JQ638923
CH/FJXM-2/2012	FJXM-2	Xiamen, Fujian	4152	1383	JX070672
CH/GD/2011	GD	Guangdong	4158	1385	JQ638915
CH/GXNN/2012	GXNN-2	Nanning, Guangxi	4158	1385	JX018179
CH/BJYQ/2011	BJYQ	Yanqing, Bejing	4161	1386	JN601048(S1); JQ305101(S2)
CH/GXQZ/2011	GXQZ	Qinzhou, Guangxi	4161	1386	JN641881(S1);JQ638913(S2)
CH/GXWP/2011	GXWP	Nanning, Guangxi	4161	1386	JN641879(S1);JQ638513(S2)
CH/HBQHD/2011	HBQHD	Qihuangdao, Hebei	4161	1386	JQ638922
CH/HLJHG/2011	HLJHG	Hegang, Heilongjiang	4161	1386	JN601046(S1);JQ638508(S2)
CH/HLJHH/2011	HLJHH	Heihe, Heilongjiang	4161	1386	JQ638916
CH/HLJHRB/2011	HLJHRB	Harbin, Heilongjiang	4161	1386	JN711456(S1);JQ638507(S2)
CH/JLCC/2011	JLCC	Chengchun, Jilin	4161	1386	JQ638920
CH/SDLY/2011	SDLY	Linyi, Shandong	4161	1386	JQ638917
CH/SDQD/2011	SDQD	Qingdao, Shandong	4161	1386	JQ638919
CH/SDRZ/2011	SDRZ	Rizhao, Shandong	4161	1386	JN671916(S1);JQ638505(S2)
CH/SH/2011	SH	Shanghai	4161	1386	JN711457(S1);JQ638511(S2)
CH/ZJHZ/2011	ZJHZ	Hangzhou, Zhejiang	4161	1386	JN641880(S1);JQ638509(S2)
CH/XJUrumqi/2011	XJUrumqi	Urumqi, Xinjiang	4161	1386	JN601047(S1);JQ638510(S2)
CH/AHHF-2/2012	AHHF-2	Hefei, Anhui	4161	1386	JX018182
CH/FJXM-1/2012	FJXM-1	Xiamen, Fujian	4161	1386	JX070671
CH/HBSN/2012	HBSN	Suning, Hebei	4161	1386	JX018183
CH/HBBD/2011	HBBD	Baoding, Hebei	4170	1389	JQ638918

**Figure 1:**
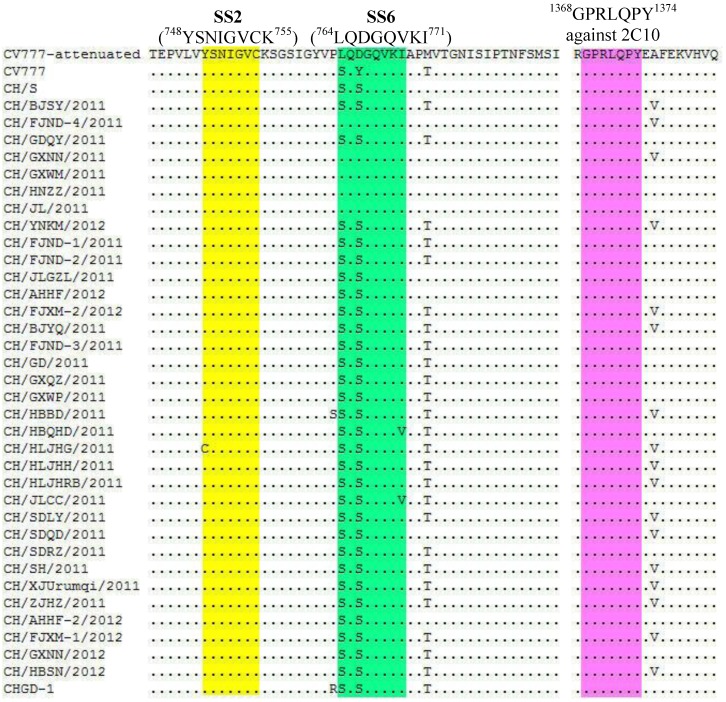
Comparison of the antigen epitopes of S proteins of field strains and reference strains. Dots indicate the amino acids are identical to those of reference strain CV777-attenuated. SS2 is in the yellow region, SS6 is in the green region and the motif against 2C10 is in the pink region.

The S proteins of recently Chinese field strains have 1,381–1,389 amino acids (aa) in length (Table 1). These consequences were due to the presences of the number of deleted or inserted amino acids that were accumulated in the N-terminus of the S protein. The antigen epitope motif (^1368^**GPRLQPY**^1374^) against 2C10, a monoclonal antibody that showed neutralizing activities against PEDV [13], the CO-26K equivalent (COE) domain (aa499–638) [14] and the epitopes SS2 (^748^**YSNIGVCK**^755^), SS6 (^764^**LQDGQVKI**^771^) [15] of S protein can induce neutralizing antibodies against PEDV. According to the result of multiple alignments of amino acid sequences, the antigen epitope motif (^1368^**GPRLQPY**^1374^) is conserved in all the Chinese field strains. The epitope SS2 is conserved in all the Chinese field strains (excluding CH/HLJHG/2011). The epitope SS6 shows 2- or 3-amino acid mutations in most field strains (Figure 1). Compared to CV777-attenuated, 7 field strains have no amino acids change in the COE domain, 27 field strains have 1-8 different amino acids in the COE domain (Table 2). 

**Table 2:** Analysis of amino acids mutations in COE domains of field strains and CV777 attenuated strain

Field strains			Amino acids in COE of CV777 attenuated strain																			Total
	500S	505I	509T	520N	526A	530H	537A	543N	546S	558T	572K	575D	576S	603G	610F	614E	615F	617S	618G	629T	632E	
BJSY, FJND-4																						0
GDQY, GXNN-1																						0
HNZZ, JL																						0
NKM																						0
GXWM																				A		1
JLGZL, HBSN					S					S				S								3
FJXM-1,GXNN-2		T								S				S								3
FJXM-2					S		V			S				S								4
GXQZ, JLCC		T								S	N			S								4
SDLY			A		S					S				S								4
FJND-1, -2					S	P				S				S				G				5
BJYQ					S	P				S				S				G				5
SDRZ		T	I							S	N			S								5
XJUrumqi		T			S	R				S				S								5
ZJHZ		T								S	N			S						I		5
AHHF, AHHF-2					S					S	N	N	T	S								6
GXWP					S					S			T	S		D	Y					6
HBBD		T							G	S				S	L				A			6
HBQHD		T				R				S	N			S				G				6
SDQD		T			S	L				S				S		D						6
GD				I	S					S			T	S		D	Y					7
HLJHG		T				R		S		S	N			S				G				7
HLJHH, HLJHRB				I	S					S			T	S		D	Y					7
SH				I	S					S			T	S		D	Y				G	8

### 2.3. Phylogenetic Analysis

**Figure 2:**
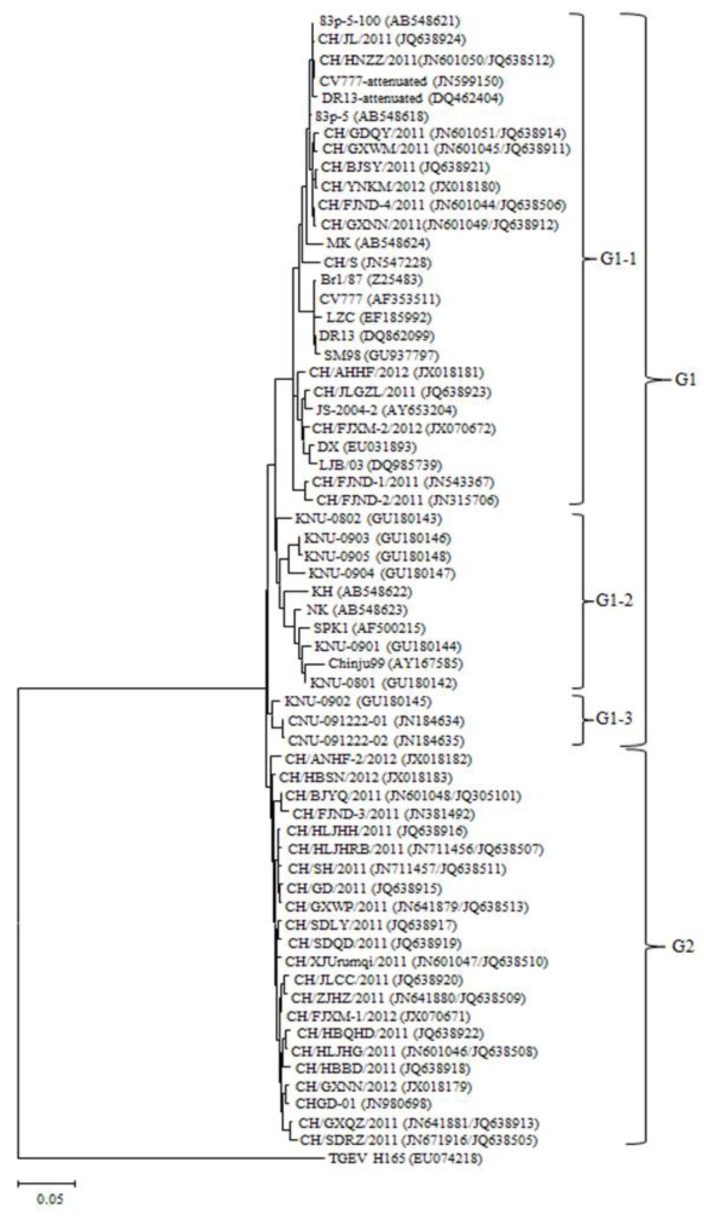
Phylogenetic analysis based on the nucleotide sequences corresponding to the full-length S gene. Putative similar regions of S gene of other distantly related coronavirus, transmissible gastroenteritis virus (TGEV), was also included in this study. The phylogenetic tree was constructed from the aligned nucleotide sequences by using the maximum likelihood method in MEGA 5.05. The scale bars indicate the number of 0.05 inferred substitutions per site. The GenBank accession numbers of recent field strains and reference strains are in the brackets.

To establish genetic relationship of the fully sequenced S genes of field strains, phylogenetic analysis was performed. The phylogenetic tree (Figure 2) indicated that all PEDV strains used in this study were divided into two groups (G1 and G2). G1 was composed of three subgroups (G1-1, G1-2 and G1-3). G1-1 comprised 27 strains including 5 classical Chinese field strains (CH/S, LZC, LJB/03, JS-2004-2, DX), 13 recently Chinese field strains, 2 European strains (CV777 and Br1/87), 2 Japanese field strains (MK and 83p-5), 2 Korean strains (SM98 and DR13), 3 attenuated strains (CV777-attenuated, DR13-attenuated and 83p-5-100). G1-2 and G1-3 were composed of 10 and 3 foreign reference strains, respectively. G2 was only composed of 22 recently Chinese field strains (including reference strains CH/FJND-3/2011, CHGD-01). In sum, 33 recently field strains were included in G1-1 and G2, and 13 field strains were included in G1-1 and 20 field strains were included in G2. 

### 2.4. Sequence Homology Analysis

We found that the nucleotide and deduced amino acid sequences of 33 field strains share 93.5%–99.9% and 92.3%–99.8% homologies with each other, respectively. The S genes of 13 recently Chinese field strains in G1-1 have 95.6%–99.9% homologies with each other, and share 96.0%–99.9%, 95.4%–96.7% and 93.6%–95.7% homologies with those of CV777-attenuated, CH/S and CHGD-1, respectively. The deduced amino acid sequences of S protein of these strains have 94.9%-99.8% homologies with each other, and share 95.9%–100%, 94.6%–96.2% and 92.7%–95.1% homologies with those of CV777-attenuated, CH/S and CHGD-1, respectively (Table 3).

The S genes of 20 recently Chinese field strains in G2 have 96.2%–99.7% homologies with each other, and share 93.6%–95.4%, 93.5%–94.2% and 97.1%–99.3% homologies with those of CV777-attenuated, CH/S and CHGD-1, respectively. The deduced amino acid sequences of these strains have 96.7%–99.7% homologies with each other, and share 92.3%–94.4%, 92.9%–93.7% and 96.6%–98.9% homologies with those of strains CV777-attenuated, CH/S and CHGD-1, respectively (Table 3).

**Table 3:** Nucleotide and deduced amino acid sequence homology of the S genes of PEDV field strains and reference strains

Groups and reference strains		Percentage identity (%) ^a^				
		G1-1	G2	CV777-attenuated	CH/S	CHGD-1
Percentage identity (%) ^b^	G1-1	95.6–99.9 ^c^ 94.9–99.8	93.5–97.1	96.0–99.9	95.4–96.7	93.6–95.7
	G2	92.3–96.5	96.2–99.7 ^d^ 96.7–99.7	93.6–95.4	93.5–94.2	97.1–99.3
	CV777-attenuated	95.9–100	92.3–94.4	***	96.7	93.6
	CH/S	94.6–96.2	92.9–93.7	96.2	***	93.3
	CHGD-1	92.7–95.1	96.6–98.9	92.7	93.0	***

^a^ Percentage of nucleotide identity (upper triangle); ^b^ Percentage of deduced amino acid identity (lower triangle); ^c^ The field strains in G1-1 show 95.6%–99.9% nucleotide identities and 94.9%–99.8% deduced amino acid identities with each other; ^d^ The field strains in G2 show 96.2%–99.7% nucleotide identities and 96.7%–99.7% deduced amino acid identities with each other.

## 3. Discussion

Since December 2010, outbreaks of viral diarrhea have been observed on most swine-breeding farms in most of the provinces of China. The clinical appearance was similar to that of PEDV infection. In this study, 79.66% (141 of 177) pig farms in 29 provinces (excluding Tibet and Hainan, China) were confirmed as infected with PEDV, and 72.27% (417 of 577) clinical samples were positive for PEDV. The results indicated that PEDV has a high prevalence rate in pig herds. TGEV was also detected by RT-PCR [16] in our lab, and the overall detection rate was 29.12% in clinical samples. Two other research groups in China reported that 40.8% (20 of 49) and 22.73% (5 of 22) sow milk samples were positive for PEDV in 2011 [5,6], and hypothesized that sow milk could represent a possible route for the vertical transmission of PEDV from sow to suckling piglets [5]. We also detected PEDV in 76.71% (56 of 73) sow milk samples in 2012. These results indicated that newborn piglets could be infected by PEDV in sow milk. 

Our previously study indicated that the S gene of CH/FJND-3/2011) has the lowest sequence homology (93.75%) with that of CH/S and is 9 nt longer than those of CV777 and CH/S [17]. A more precise study with the full-length S genes of recently Chinese field strains will help to best understand the genetic relationships among prevailing PEDV in China. In this study, the entire S genes of 33 representative Chinese field strains during 2011–2012 were determined and compared with those of CV777-attenuated, CH/S and CHGD-1. 

The S genes of attenuated strains (CV777-attenuated, DR13-attenuated, 83p-5-100) are 4149 nt in length [18,19], however those of classical virulent strains are 4152 nt in length, such as CV777 [20], Br1/87 [21] and Chinese classical strain CH/S [22]. The S genes of 33 recently field strains show the diversity in length. The S genes of 13 recently field strains in G1-1 are shorter than those of classical strains in length because of the nucleotides deletion, however, the S genes of 20 recently field strains in G2 are longer than those of classical strains in length because of the nucleotides insertion and deletion. 

The unique characteristic of 20 field strains in G2 in the present study was the presence of the nucleotides insertion and deletion in the S genes. The alignment of S genes reveals that one domain which locates within the 5' most 1100 nt of the S gene exhibited an increased divergence compared to the remaining part of the sequence. The insertion and deletion regions are mainly clustered in this region. Furthermore, the largest number of nucleotide differences is also clustered in this domain. Most of the diversity between the recent field strains in G1-1 and in G2 is located within the 1100 nt-domain. The phenomenon is also found in the S gene of CH/FJND-3/2011 [17]. 

Our findings showed the S genes of 13 field strains in G1-1 exhibited higher homologies with those of CH/S and CV777-attenuated than with that of CHGD-1. However, the S genes of 20 field strains in G2 exhibited higher sequence identities with that of CHGD-1 than with those of CH/S and CV777-attenuated. These results further indicated the heterogeneity in S genes of PEDV field strains prevailing in China. 

According to the phylogenetic analyses, the recently Chinese field strains were divided into two distinct clusters. 13 field strains and reference strains including Korean field strains, Japanese strains, PEDV-attenuated strains and Chinese classical strains were determined to belong to G1, whereas 20 field strains and 2 reference strains (CH/FJND-3/2011, CHGD-1) were clustered together within G2. These data demonstrate that prevalent PEDV field strains in China have two types (classical strains and variant strains), and most field strains are variant strains. These variants are more closely related to each other and to other previously identified Chinese variants CH/FJND-3/2011 and CHGD-1 rather than Chinese classical strains (CH/S, DX, JS-2004-2, LJB/03, LZC) and attenuated strains.

The antigen epitope motif (^1368^GPRLQPY^1374^) [13] and the COE domain (aa499-638) [14] of S protein can induce neutralizing antibodies against PEDV. According to the alignment of amino acid sequences of S proteins, the antigen epitope motif (^1368^GPRLQPY^1374^) is conserved in all the recent field strains. The COE domain consists of 140 aa. Compared to CV777-attenuated, 27 field strains have 1-8 different aa, 7 field strains have no different aa. A previously study reported that three of these mutations in the recent isolates influenced the hydrophobicity of the S protein as compared with that for CV777 [5]. 

## 4. Experimental

### 4.1. Clinical Samples Detection

A total of 577 samples (504 small intestines of dead neonatal piglets and 73 sow milk) were collected from 177 farms in 29 provinces from February 2011 to November 2012. The collection procedures of milk were as follows. The sows’ teats were washed three times with sterile physiological saline. The workers milked the teats with sterile gloves, and took the middle section of the milk in 1.5mL sterile microcentrifuge tubes. The milk samples were sent to our lab with ice bags or dry ice. All the samples were initially detected by reverse transcription-polymerase chain reaction (RT-PCR) using previously described methods [23]. The animal experiments were approved by Harbin Veterinary Research Institute and performed in accordance with animal ethics guidelines and approved protocols. The approval number of Animal Ethics Committee is Heilongjiang-SYXK-2006-032.

### 4.2. Sequencing of S Gene

To obtain the full-length S gene, four primers (S1-1U, 5'- ATCGTCAGAGGCATTTTTAA-3'; S1-1L, 5'-ATCCATCACCATTAAACGAA-3'; and S1-2U, 5'- ATGTTGTGTTAGGCTTGTTG-3'; S1-2L, 5'- CACTAACAGGCGTGTTGTAA-3') and three primers (S2-U, 5'- CTGATTCTGGACAGTTGTTA-3'; S2-1L, 5'- TTGGACAGCATCCAAAGACA-3'; S2-2L, 5'- CTTCGAGACATCTTTGACAA-3') were designed and synthesized according to the corresponding region of CV777 (AF353511) to amplify S1 domain and S2 domain by PCR or nested-PCR, respectively. The complementary DNA (cDNA) of 33 representative field strains was synthesized using S2-2L as the reverse transcription primer according to the previously described methods [23].

PCR was carried out in a two-step reaction, first with a pair of primers flanking the region to be amplified and then using a pair of primers within the amplified sequence in a total volume of 25 µL. Exactly, 1µL (10 ng) cDNA was mixed with a reaction mixture containing 12.5 µL 2× EmeraldAmp^TM^ PCR Master Mix (TaKaRa, Dalian, China), 0.5 µL each specific primer (10 µM), and 10.5 µL sterile deionized water. The first round of amplification was performed under the reaction conditions (pre-denaturation at 94 °C for 5 min followed by 30 cycles of denaturation at 98 °C for 10 s, annealing at 50 °C for 30 s, extension at 72 °C for 3 min, and a final extension at 72 °C for 7 min). The second round of amplification was performed: a 100-fold dilution in distilled water of the first PCR products were used as the template under the reaction conditions of the first round of amplification (pre-denaturation at 94 °C for 5 min followed by 25 cycles of denaturation at 98 °C for 10 s, annealing at 50 °C for 30 s, extension at 72 °C for 2.5 min, and a final extension at 72 °C for 7 min).

PCR products were excised from 1.0% agarose gels, extracted using QIAquick® Gel Extraction Kit (QIAGEN GmbH, Hilden, German), cloned into a T-tailed vector, pMD18-T (TaKaRa, Dalian, China) and transformed using JM109 competent cells (TaKaRa, Dalian, China). Three to five recombinant DNA clones of each PEDV strains were sequenced by Beijing Genomics Institute (Beijing, China). 

### 4.3. Multiple Alignments and Phylogenetic Analysis

The sequences of full-length S genes of 33 field strains (Table 1) and 30 reference strains (Table 4) were used in sequence alignments and phylogenetic analysis. The multiple sequence alignments, nucleotide and amino acid sequences divergences were analyzed by Jotun Hein method in the software program MegAlign [24]. Phylogenetic tree was constructed from the aligned nucleotide sequences by the maximum likelihood method in MEGA 5.05 program [25].

**Table 4:** Reference strains used in this study

Reference strains	Countries	S genes (nt)	S proteins (aa)	Accession no.
CV777	Belgium	4152	1383	AF353511
Br1/87	British	4152	1383	Z25483
CH/S	China	4152	1383	JN547228
LZC	China	4152	1383	EF185992
JS-2004-02	China	4152	1383	AY653204
DX	China	4152	1383	EU031893
LJB/03	China	4152	1383	DQ985739
CV777 attenuated	China	4149	1382	JN599150
CHGD-1	China	4158	1385	JN980698
CH/FJND-3/2011	China	4161	1386	JQ282909
Chinju99	South Korea	4152	1383	AY167585
DR13	South Korea	4152	1383	DQ862099
DR13 attenuated	South Korea	4149	1382	DQ462404
SM98	South Korea	4143	1380	GU937797
SPK1	South Korea	4161	1386	AF500215
KNU-0801	South Korea	4161	1386	GU180142
KNU-0802	South Korea	4161	1386	GU180143
KNU-0901	South Korea	4161	1386	GU180144
KNU-0902	South Korea	4161	1386	GU180145
KNU-0903	South Korea	4161	1386	GU180146
KNU-0904	South Korea	4161	1386	GU180147
KNU-0905	South Korea	4161	1386	GU180148
CNU-091222-01	South Korea	4161	1386	JN184634
CNU-091222-02	South Korea	4161	1386	JN184635
83p-5	Japan	4152	1383	AB548618
83p-5-100	Japan	4149	1382	AB548621
KH	Japan	4164	1387	AB548622
NK	Japan	4176	1391	AB548623
MK	Japan	4152	1383	AB548624
TGEV H165	China	4347	1448	EU074218

## 5. Conclusions

This study confirmed that PEDV has a high detection rate in pig herds in China. The sequence analysis indicated the S genes of recent field strains are heterogeneous. The phylogenetic analysis indicated that there are two types of PEDV strains (classical strains and variant strains) prevailing in China. The sequence information in the current study would lead to a better understanding of the genetic diversity among recently Chinese PEDV strains and contribute to the development of more effective vaccines. The future work should focus on the variants of PEDV in China and develop novel variant strain–based vaccines to treat the current outbreak in China. 
